# RPL27A is a target of miR-595 and may contribute to the myelodysplastic phenotype through ribosomal dysgenesis

**DOI:** 10.18632/oncotarget.10293

**Published:** 2016-06-25

**Authors:** Heba A. Alkhatabi, Donal P. McLornan, Austin G. Kulasekararaj, Farooq Malik, Thomas Seidl, David Darling, Joop Gaken, Ghulam J. Mufti

**Affiliations:** ^1^ Department of Haematological Medicine, King's College London School of Medicine, London, UK; ^2^ Center of Excellence in Genomic Medicine Research, King Abdul Aziz University, Jeddah, Saudi Arabia; ^3^ Department of Hematology, King's College Hospital, London, UK

**Keywords:** myelodysplasia, ribosome, microRNA, haemopoiesis, chromosome

## Abstract

We investigated the functional consequences following deletion of a microRNA (miR) termed miR-595 which resides on chromosome 7q and is localised within one of the commonly deleted regions identified for Myelodysplasia (MDS) with monosomy 7 (−7)/isolated loss of 7q (7q-). We identified several targets for miR-595, including a large ribosomal subunit protein RPL27A. RPL27A downregulation induced p53 activation, apoptosis and inhibited proliferation. Moreover, p53-independent effects were additionally identified secondary to a reduction in the ribosome subunit 60s. We confirmed that RPL27A plays a pivotal role in the maintenance of nucleolar integrity and ribosomal synthesis/maturation. Of note, RPL27A overexpression, despite showing no significant effects on p53 mRNA levels, did in fact enhance cellular proliferation. In normal CD34+ cells, RPL27A knockdown preferentially blocked erythroid proliferation and differentiation. Lastly, we show that miR-595 expression appears significantly downregulated in the majority of primary samples derived from MDS patients with (−7)/(7q-), in association with RPL27A upregulation. This significant downregulation of miR-595 is also apparent when higher risk MDS cases are compared to lower risk cases. The potential clinical importance of these findings requires further validation.

## INTRODUCTION

MicroRNAs (miRNAs) are short non-coding RNAs which play pivotal roles in a diverse range of cellular processes [1]. As current miRNA target identification assays are often associated with a lack of sensitivity and specificity, this led to our development of a novel assay to identify potential miRNA targets [2]. This assay relies upon functional activity rather than depending solely on computational algorithms addressing homology or binding to a putative target [3–5]. Furthermore, this methodology can aid identification of targets that are downregulated by mRNA cleavage or translation inhibition.

We focused on miRNAs localised to chromosome 7, in particular *miR-595* at 7q36.3. This miRNA is localised within one of the commonly deleted regions (CDR) identified for MDS with monosomy 7 (−7)/isolated loss of 7q (7q-) [6–9]. We show, for the first time, that *RPL27A*, encoding a ribosomal subunit protein, is a target for *miR-595*.

**Figure 1:**
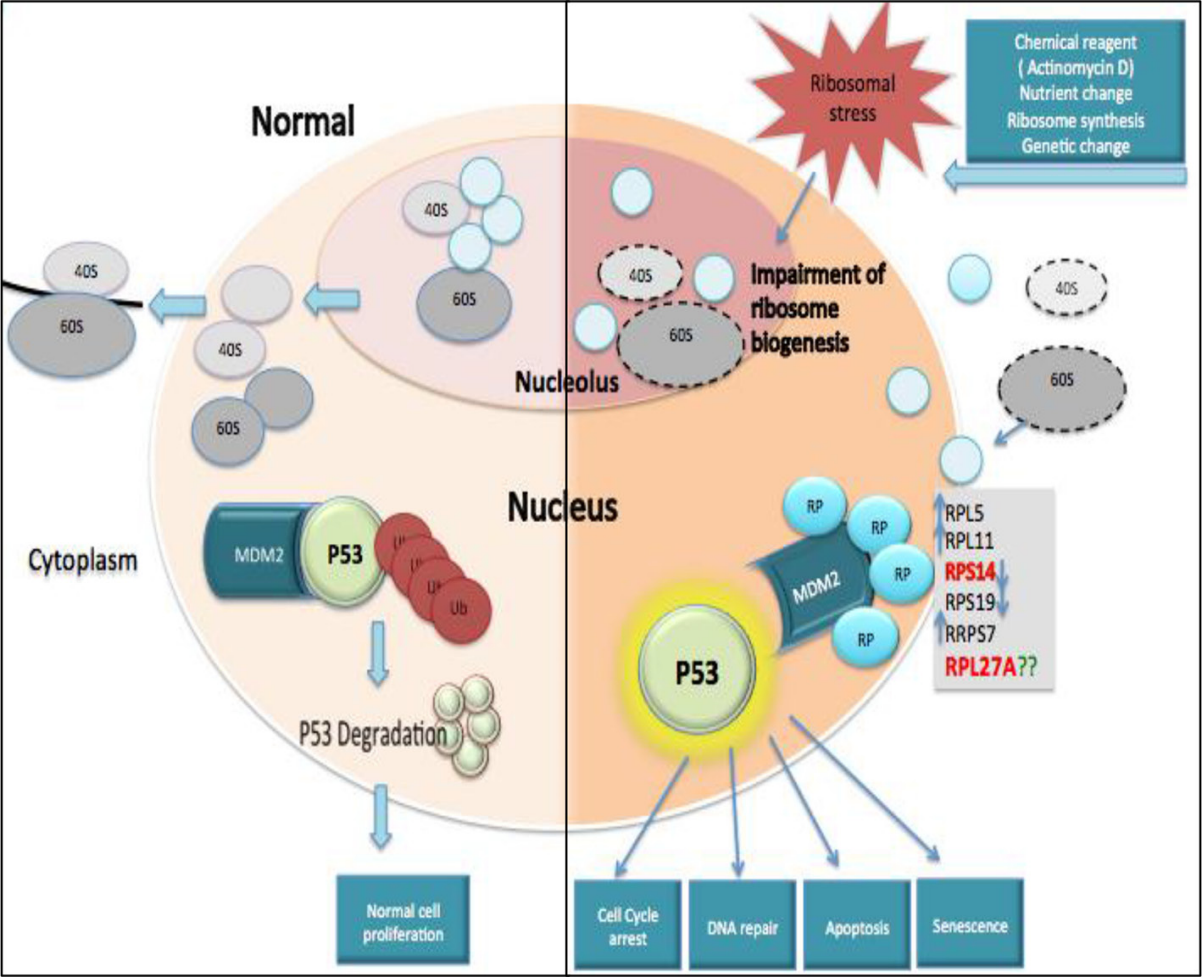
Ribosomal protein-MDM2-p53 interactions Under steady state conditions, ribosome biogenesis occurs within the nucleolus and ribosome subunits are subsequently exported to the cytoplasm to facilitate formation of mature ribosomes and initiation of mRNA translation. MDM2 can mediate attachment of ubiquitin (Ub) molecules to p53, inducing p53 degradation and permitting normal cell proliferation. In contrast, ribosomal stress can impair ribosome biogenesis and disrupt ribosome assembly. This causes a release of free ribosomal proteins (RP), which interact with MDM2 and block its interaction with p53, resulting in p53 accumulation. Subsequently, activated p53 may cause cell cycle arrest, apoptosis, deregulated DNA repair or senescence.

It is well established that impaired ribosome biogenesis can induce nuclear stress. This phenomenon can be associated with the development of clinical entities collectively known as “ribosomopathies” which include several congenital disorders and bone marrow failure syndromes, such as Diamond Blackfan Anaemia (DBA), Shwachman-Diamond syndrome (SDS), Dyskeratosis Congentia (DC) and Cartilage-Hair Hypoplasia (CHH) syndrome, in addition to the acquired MDS subtype ‘5q- syndrome’ [10, 11]. More recently, it has also been recognised that ribosomal stress can induce p53 accumulation, although the mechanisms underlying this phenomenon remain complex (Figure 1). Marechal *et al.* were the first to demonstrate the complex interaction between RPL5, p53 and MDM2 [12]. Further studies have demonstrated that reduced expression of a range of ribosomal proteins, including RPS6, RPL22, RPL24, RPS19, RPS14 and RPL23, can all increase p53 levels whereas reductions in RPL11 and RPL5 actually decrease p53 levels [13–20]. In contrast, the overexpression of RPL5, RPL26 and RPL11 and other ribosomal proteins, induced p53 expression [19–23]. Uniquely, both reductions and overexpression of RPL23 and RPS14 augment p53 expression, highlighting the complex interaction of these ribosomal proteins and the p53 axis [18–24]. In this study, we focused on validating RPL27A as a novel target of miR-595. We investigated the effects of RPL27A knockdown on p53 activation, ribosome synthesis and maturation. Lastly, we investigated the expression of miR-595 in a cohort of patients with MDS.

## RESULTS

### Identification and validation of RPL27A as a target of miR-595

Our novel assay is based on the directional cloning of a 3′UTR cDNA target ID library (Sigma, MREH01) derived from 10 different human tissues and 10 human cell lines, representing 16,923 unique genes, cloned downstream of a TKzeo fusion gene in plasmid p3′TKzeo conferring zeocin resistance and Ganciclovir sensitivity. The breast carcinoma MCF7 cell line, lacking miR-595, was hence transfected with this cDNA library and underwent zeocin selection. Expanded zeocin resistant cells underwent infection with either a pBabePuro vector expressing *miR-595* or empty vector. After 48 hours, cells underwent puromycin selection and subsequent ganciclovir (GCV) counter-selection. Surviving colonies were expanded, genomic DNA isolated and PCR amplified using vector specific primers. Supplementary Figure S1 displays one such amplification. Candidate amplicon bands were present in multiple independent samples and hence represented putative *miR-595* targets. These bands of interest underwent purification and sequencing. A BLAST sequence search displayed a high degree of homology for two different transcripts; ribosomal protein L27A (*RPL27A*; 500 bp) and heat shock protein 14 (*HSPA14*; 800 bp). We focused on validation of *RPL27A* as a target for *miR-595* and establishing the biological function of *RPL27A*.

**Figure 2:**
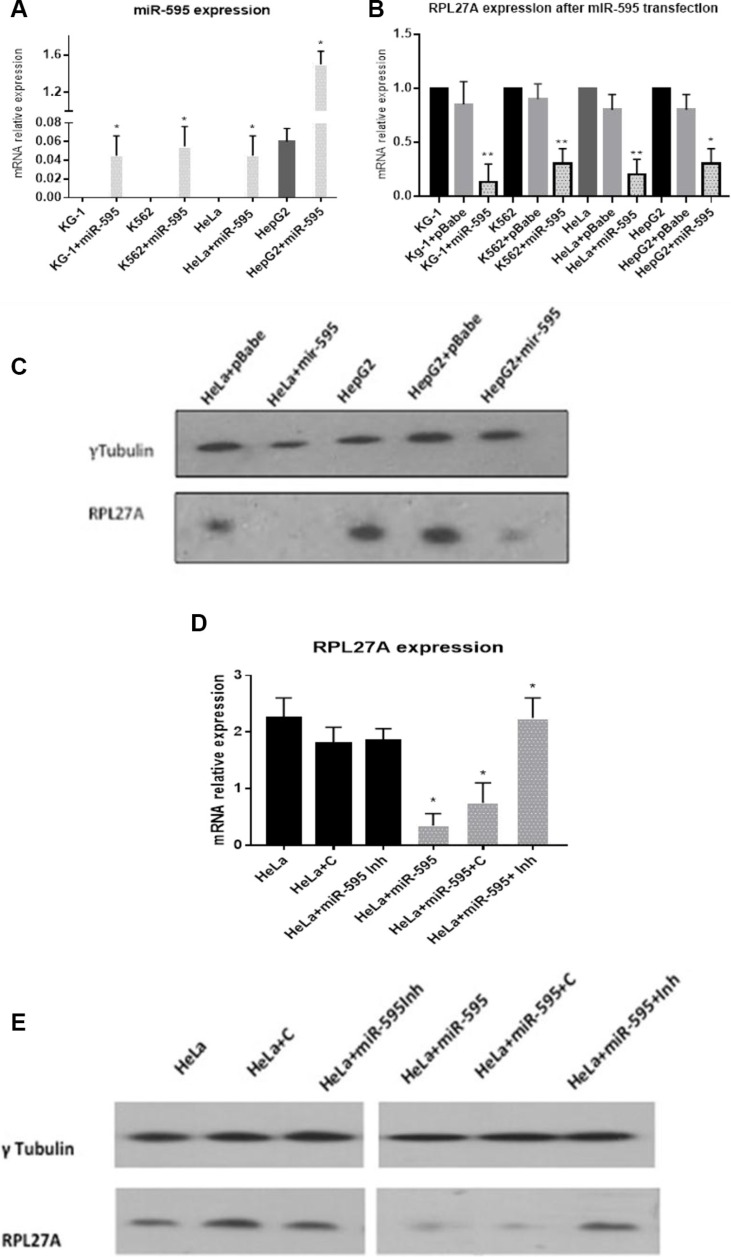
Validation of RPL27A as a target for *miR-595* by qRT-PCR and western blot analysis Endogenous RPL27A mRNA and protein levels were determined in untransfected, pBabePuro empty vector (pBabe), and pBabePuro-miR-595 (miR-595) transfected cells. *miR-595* expression levels were determined in untransfected and *miR-595* transfected KG-1. K562, HeLa and HepG2 cells 48 hrs after the miRNA transfection (**A**). To investigate if miR-595 overexpression downregulates *RPL27A* expression, *RPL27A* mRNA was quantified by qRT-PCR in untransfected cells, cells transfected with empty vector and cells transfected with *miR-595*. Downregulated expression of *RPL27A* occurred in all cell lines overexpressing *miR-595*, in contrast to cells transfected with pBabe empty vector and untransfected cells. Results represent three independent experiments; bars display the mean ± standard error of the mean (SEM) (**B**). HeLa and HepG2 cells were transfected with empty vector or *miR-595*. RPL27A protein expression is downregulated in HeLa and HepG2 cells expressing *miR-595* compared to control. Tubulin was used as a loading control (**C**). HeLa cells which did not express *miR-595* were transfected with either hairpin control inhibitor (HeLa+C) or *miR-595* inhibitor (HeLa+ miR-595Inh). HeLa cells expressing *miR-595* were also transfected with hairpin control or *miR-595* inhibitor. Upregulated *RPL27A* mRNA and protein expression was evident in *miR-595* expressing HeLa cells which underwent transfection with the *miR-595* inhibitor compared to controls (**D**, **E**).

Validation experiments were performed in the HeLa, HepG2, KG-1 and K562 cell lines. Each underwent transfection with either pBabepuro-*miR-595* or empty vector followed by puromycin selection. Cells were screened for *miR-595* expression by quantitative – Reverse Transcriptase PCR (qPCR) and demonstrated significantly increased *miR-595* expression compared to wild-type (Figure 2A). Following *miR-595* overexpression, *RPL27A* mRNA levels were decreased by 83% in KG-1 cells, 73% in K562 cells, 88% in HeLa cells and 55% in HepG2 cells respectively compared to controls (Figure 2B). RPL27A protein levels in both HeLa and HepG2 cells were also downregulated (Figure 2C). Lastly, HeLa wild-type cells and *miR-595*-transfected HeLa cells underwent additional transfection with a hairpin inhibitor directed against *miR-595* (Miridian) and a control hairpin inhibitor (Dharmacon) and analysed for RPL27A mRNA and protein expression (Figure 2D). Wild-type HeLa cells showed no significant alteration in RPL27Aexpression levels following inhibitor transfection, whereas HeLa cells transfected with *miR-595* showed reduced expression of RPL27A. This reduced RPL27A expression could be reversed with the *miR-595* hairpin inhibitor (Miridian), resulting in a 4-5-fold upregulation of RPL27A mRNA transcripts and protein expression.

### Decreased expression of *RPL27A* causes p53 activation

We examined the effects of reduced *RPL27A* expression in HCT-116, HCT-116-p53^−/−^, K562 and HEL cell lines and compared these to the already well-established effects induced by *RPS14* and *RPL5* downregulation. Two different lentivirus short-hairpin (sh) RNAs were chosen, *RPL27A*-sh2 and *RPL27A*-sh4, which decreased expression by 80% and 40% respectively at mRNA and protein levels. Efficient knockdown of both *RPS14* and *RPL5* via lentiviral shRNA was also achieved (Supplementary Figure S2A–S2D).

**Figure 3:**
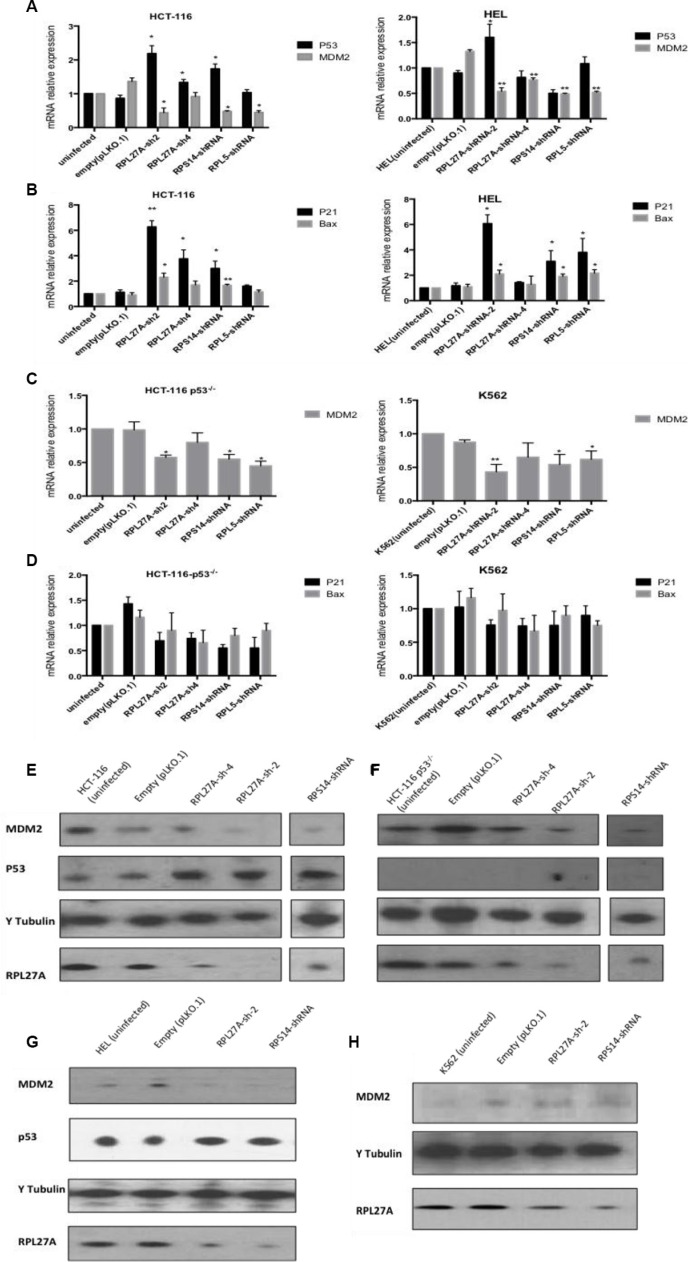
Decreased expression of *RPL27A* activates the p53 pathway HCT-116 and HEL cell lines dually express *MDM2* and *p53* mRNA. RPL27A and RPS14 depletion significantly augmented expression of *p53* mRNA in HCT-116 cells compared to controls. *RPL27A-sh2RNA*, *RPS14-shRNA* and *RPL5-shRNA* reduced *MDM2* expression in both cell lines (**A**). Increased expression of the p53 target genes *p21* and *Bax* was induced by *RPL27A* and *RPS14* depletion in HCT-116 and HEL cells (**B**). In the HCT116 p53^−/−^ and K562 cell lines, MDM2 expression was reduced following *RPL27A-shRNA2*, *RPS14-shRNA* and *RPL5-shRNA* infection but no significant effects were noted for *p21* or *Bax* expression. Bars display the mean results from three independent experiments ± SEM (*p* values were determined using two tail student *t*-test *p = < 0.05; **p = < 0.005)) (**C**, **D**). Protein expression of p53, MDM2 and RPL27A was determined following RPL27A-sh2 or RPS14-shRNA mediated knockdown. Tubulin was used as a loading control. In the HCT-116 and cell lines, depletion of *RPL27A* and *RPS14* induced p53 expression and reduced MDM2 (**E**, **G**). In the HCT-116 p53^−/−^ cell line, depletion of *RPL27A* and *RPS14* reduced MDM2 expression (**F**). No significant reduction in MDM2 protein expression was identified compared to empty vector following infection with either *RPL27A* shRNA and *RPS14* shRNA in K562 cells (**H**). In all cell lines, infection with *RPS14* shRNA, caused a reduction in RPL27A expression. Results are representative of 3 independent experiments.

In the p53-expressing HCT-116 cell line, *RPL27A*-sh2 and -sh4 mediated knockdown resulted in a significant increase in *p53* mRNA levels compared to control (*p* = 0.005 and *p* = 0.021 respectively). For the p53 expressing HEL cells, an increase in *p53* expression was only observed following *RPL27A*-sh2 (*p* = 0.027), *RPS14* knockdown resulted in significant *p53* upregulation (*p* = 0.0149) whereas *RPL5* depletion did not induce any significant changes (Figure 3A).

MDM2 is a pivotal feedback regulator of the p53 pathway. Depletion of *RPL27A, RPS14* and *RPL5* resulted in reduced MDM2 expression at both the transcriptional and post-transcriptional level in all cell lines except in K562 cells, where *RPL27A*-sh2 induced significant reductions at mRNA level only (Figure 3A and 3C). Co-immunoprecipitation assays demonstrated that endogenous RPL27A interacts with endogenous MDM2 and RPL5 in HCT-116 cells (Supplementary Figure S3).

To investigate if induction of *p53* expression resulted in transcriptional activation, expression of the p53 targets *p21* and *Bax* was determined. In HCT-116 cells, both *p21* and *Bax* mRNA levels were significantly increased following both *RPL27A*-shRNA and *RPS14*-shRNA-mediated downregulation. In contrast, *RPL5* knockdown did not induce significant changes. In HEL cells, *RPL27A*-sh2, *RPS14* shRNA and *RPL5* shRNA induced significant increases in both *bax* and *p21* expression. As expected, for the null p53 cell lines, no changes in *p21* and *Bax* expression were identified, indicating that *p21* and *Bax* induction occurred in a p53-dependent fashion (Figure 3B and 3D). Both *RPL27A*-sh2 and *RPS14* shRNA, compared to controls, increased p53 protein expression in HCT-116 and HEL cells. Interestingly, knockdown of *RPS14* also resulted in a decreased expression of RPL27A (Figure 3E). Of note, RPL27A overexpression in HCT-116 cells showed no significant effect on *p53* mRNA levels or alteration in the cell cycle profile but did induce proliferation (Supplementary Figure S4A–S4D).

### Decreased expression of *RPL27A* induces apoptosis and inhibits proliferation in a p53 independent manner

**Figure 4:**
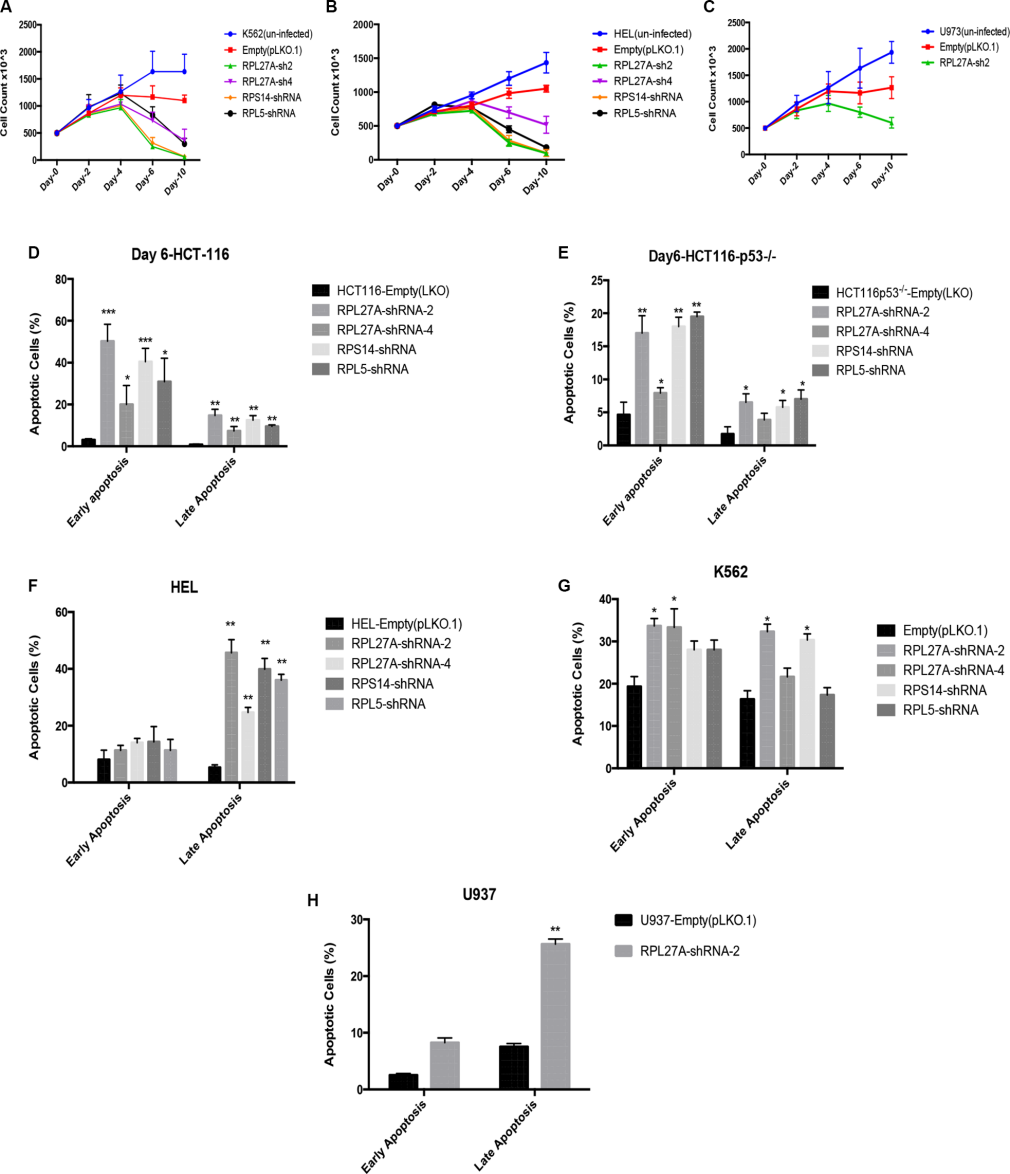
Reduction in *RPL27A* decreases cell proliferation and induces apoptosis Proliferation was assessed by cell enumeration at 48-hour intervals. Significant reductions in K562 and HEL cells were determined by day 4 post-infection with *RPL27A*-sh2, *RPL27A*-sh4, *RPS14* shRNA and *RPL5*-shRNA compared to controls (**A**, **B**). Although attenuated proliferation was also noted in the U937 cells following *RPL27A*-sh2, this was less marked than in the other cell lines examined (**C**). Error bars represent the mean ± SEM from three independent experiments. Apoptosis was assessed via flow cytometric assessment of Annexin V and 7-ADD expression in HCT-116, HCT-116 p53^−/−^, K562 and HEL cells at day 6 following infection with empty vector, both *RPL27A* shRNAs, *RPS14* shRNA and *RPL5* shRNA. Compared to controls, RPL27A depletion induced significant apoptosis in all 4 cell lines (**D**–**G**). Similarly, knockdown of RPS14 and RPL5 induced significant apoptosis. In the U937 cell line, compared to empty vector, RPL27A-sh2 infection significantly increased the percentage of cells undergoing apoptosis but to a lesser degree than observed in the other cell lines (**H**). Columns display the mean results from three independent experiments ± SEM (*p = < 0.05; **p = < 0.005)).

Initially, proliferation assays were performed in both p53-expressing and p53-null cells to determine effects of *RPL27A* depletion (Figure 4A–4B). In both K562 and HEL cells, a significant reduction in cell numbers was detected by day 4, and increased over time, following *RPL27A* knockdown by *RPL27A*-sh2. This was also the case for *RPS14* and *RPL5* knockdown. *RPL27A*-sh4 infection, however, displayed less effect on cell numbers. Unexpectedly, we did not observe significant differences in cell death between p53 expressing HEL cells and p53 null K562 cells. To establish if this was truly a p53-independent effect, we repeated knockdown experiments with *RPL27A*-sh2 in the p53 null cell line U937. Again we noted a decrease in cell numbers compared to control, although this was not lethal (Figure 4C).

Apoptosis assays using Annexin-V/7-AAD staining at Day 6 following shRNA infection were performed (Figure 4D–4H). HCT-116 infection with *RPL27A*-sh2 induced significant increases in cells at the early stage of apoptosis (55%), as was the case with *RPS14* (40%) and *RPL5* (35%) knockdown. Of note, *RPL27A*-sh4, which induces < 50% *RPL27A* knockdown, only led to a 25% increase in apoptotic cells. The HCT-116-p53^−/−^ cell line also displayed significant increases in early (range 15–20%) and late stages of apoptosis (8–10%). For HEL cells, a significant increase in the late stage of apoptosis was observed for all shRNAs (40–50%). However, in p53 null K562 cells, depletion of *RPL27A* and *RPS14* led to an increase in apoptotic cells by an average of 15–20% at both early and late stages. Lastly, U937 cells demonstrated a significant, but less marked, induction in apoptosis following *RPL27A*-sh2 infection.

### *RPL27A* deficiency induces a reduction in ribosomal 60S and causes nucleolar disruption

**Figure 5:**
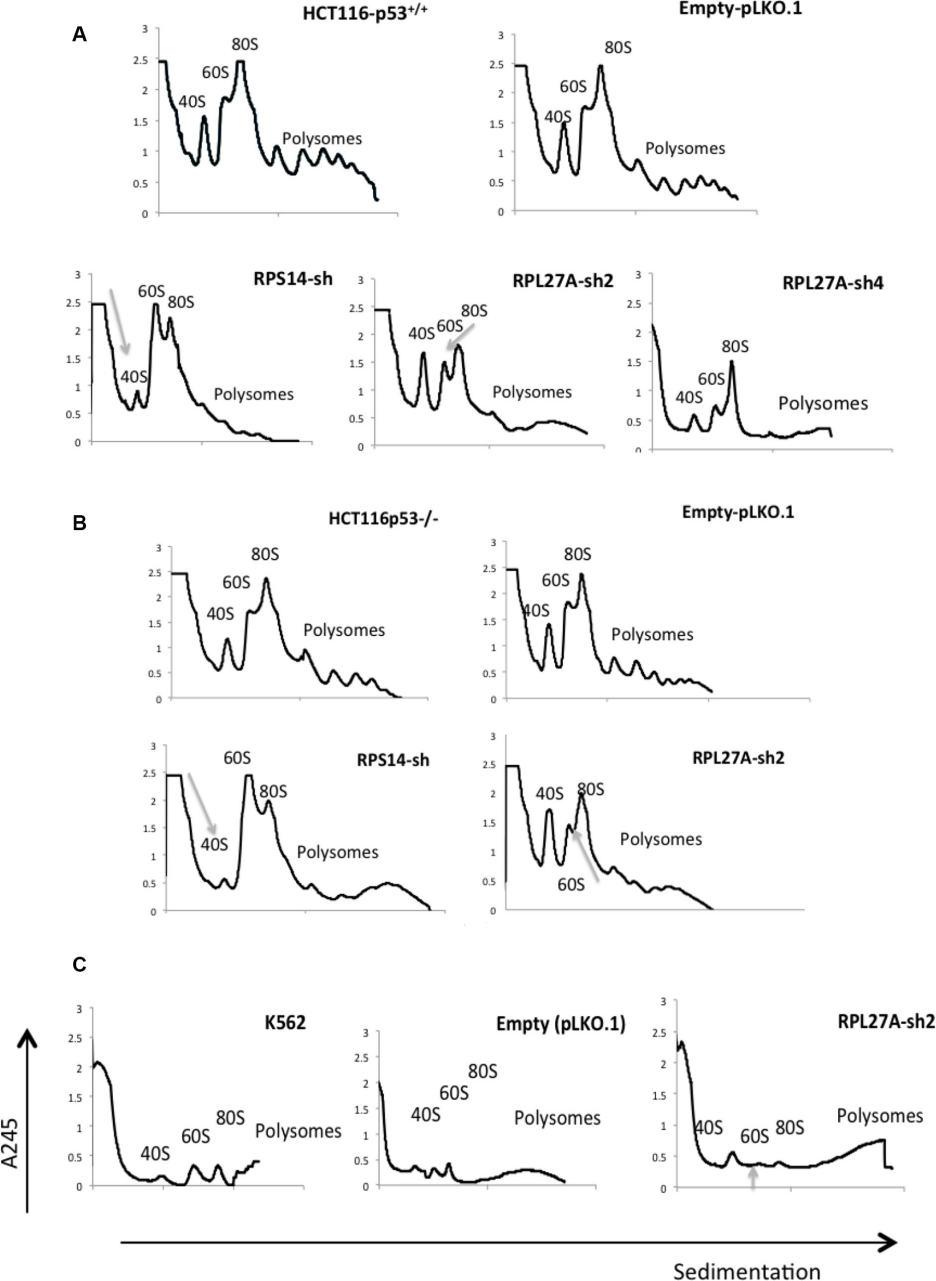

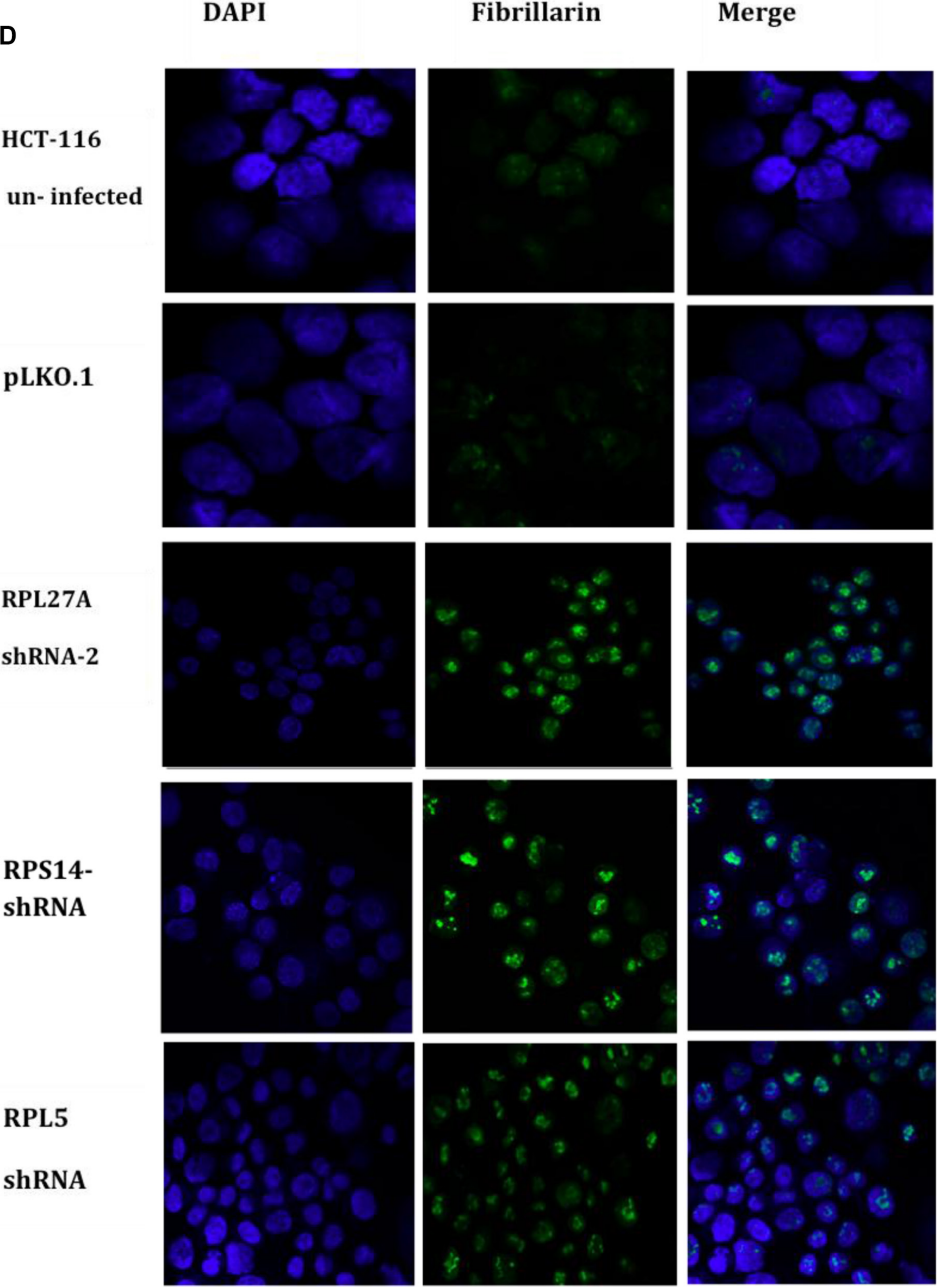
Depletion of *RPL27A* causes defects in 60S subunit maturation Polysome profiling, utilising sucrose gradient fractionation, was performed in cells post infection with lentivirus-expressing *RPL27A* shRNAs, *RPS14* shRNA and empty vector pLKO.1 respectively. Polysomes were detected at A_245_ wavelength using a UV monitor at day 6 post infection and ribosomal native subunits 40S, 60S and 80S are indicated. In HCT-116 and HCT-116 p53^−/−^ cells, *RPL27A* depletion induced a significant reduction in the 60S subunit relative to control. As predicted, *RPS14* shRNA reduced the 40S subunit compared to the relative control (**A, B**). *RPL27A* shRNA also reduced the 60S subunit in the K562 cell line (**C**). Results are representative of duplicate experiments. HCT-116 cell controls, post-infection with pLKO.1 empty vector or the ribosomal protein directed shRNAs as indicated, were fixed on slides and stained with an anti-fibrillarin antibody (green: nucleolar marker) on day 6. The nucleus was stained with DAPI (blue). Slides were analysed using a 40× objective Zeiss LSM780 confocal microscope and ZEN 2010 software. Results are representative of duplicate experiments. Disruption of nucleolar fibrillarin staining was evident in cells infected with shRNAs targeting the ribosomal proteins as indicated when compared to pLKO.1 empty vector (**D**).

Polysome profiling was performed to establish if *RPL27A* deficiency induced ribosomal defects. Profiling was performed in HCT-116, HCT-116-p53^−/−^ and K562 cells infected with empty vector, *RPL27A*-sh2 or *RPS14* shRNA respectively. Downregulation of *RPL27A* resulted in a reduction of the 60S subunit and slight increase in the 40S subunit in all three-cell lines (Figure 5). Of note, *RPL27A*-sh4 was examined in HCT-116 cells where it induced a slight reduction in the 60S subunit only.

Next, nucleolar staining was performed. HCT-116 and HCT-116 p53^−/−^ cells were infected with empty vector, *RPL27A*-sh2, *RPS14*-shRNA or *RPL5*-shRNA, underwent puromycin selection and subsequently were collected, fixed and stained with anti-fibrillarin antibody. Depletion of *RPL27A*, *RPS14* and *RPL5* resulted in abnormal dispersion of fibrillarin in the nucleolus (Figure 5). These characteristics may in part explain the p53-independent effects observed following *RPL27A* depletion.

### *RPL27A* depletion induces early p53 dependent effects

**Figure 6:**
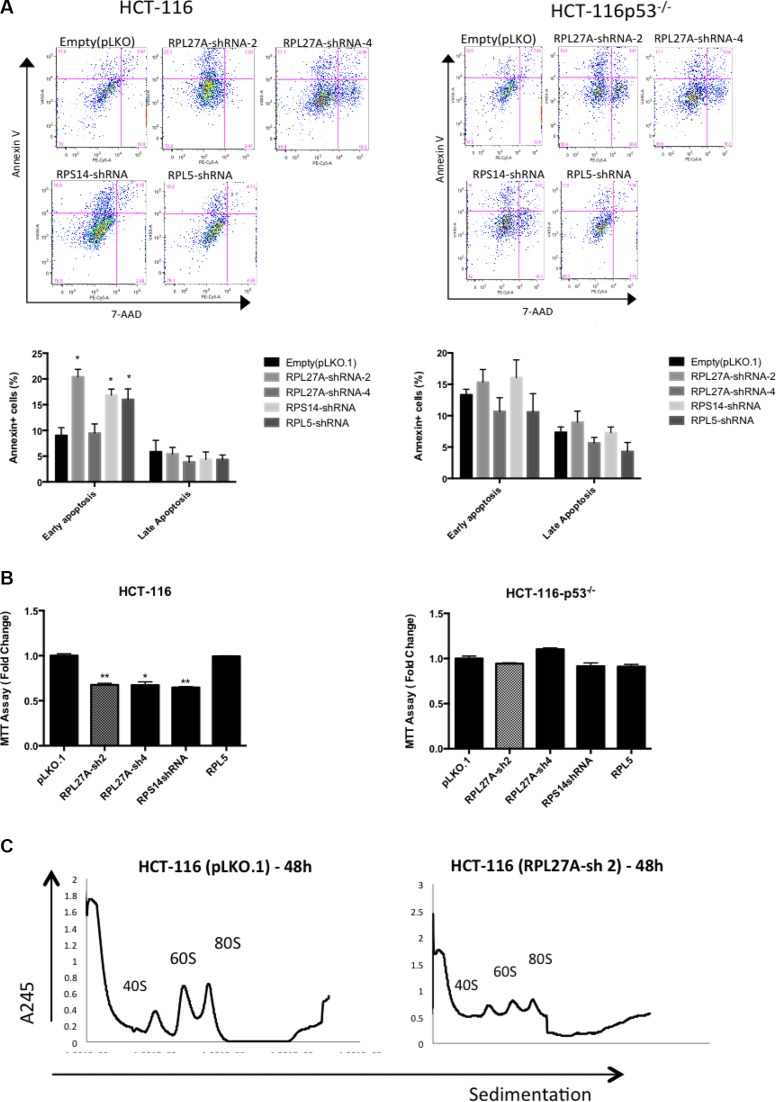
RPL27A deficiency results in early p53-dependent apoptotic defects HCT-116 and HCT-116 p53^−/−^ cells were infected with control empty vector or shRNAs directed against *RPL27A*, *RPS14* and *RPL5* respectively and collected for analysis at 48-hours post infection. For flow cytometric analysis, cells were stained with Annexin and 7-AAD. Cells in the upper left quadrant indicate Annexin positive, early apoptotic cells. Cells in the upper right quadrant indicate Annexin-positive/7-AAD-positive, late apoptotic cells. Compared to empty vector, RPL27A-sh2, RPS14 shRNA and RPL5 shRNA significantly induced apoptosis in the p53 containing HCT-116 cells only (**A**). Column diagrams represent mean % of apoptotic cells from three independent experiments ± SEM. (*p < 0.05; **p < 0.005). MTT assays were performed to delineate effects on cell viability (**B**). A significant reduction in cell viability was evident following RPL27A-sh2, -sh4 and RPS14 shRNA in HCT-116 cells only and not in the HCT-116 −/− cell line. Results are expressed as relative fold change mean ± SEM and are representative of three independent experiments (*p < 0.05; **p < 0.005). Polysome profiling (using sucrose gradient fractionation) was performed in HCT-116 cells infected with either RPL27A-sh2 or empty vector only. Polysomes were detected at A_245_ using an ultraviolet monitor. RPL27A depletion reduced the 60S subunit compared with relevant controls; experiments performed in duplicate (**C**).

To investigate time-dependent effects following *RPL27A* depletion, we collected samples for analysis from HCT-116 and HCT-116 p53^−/−^ cells at 48 hours post-infection with *RPL27A*-sh2, *RPS14* shRNA, *RPL5* shRNA or control. *RPL27A*-sh2, *RPS14* shRNA and *RPL5* shRNA infection induced p53-dependent apoptosis. In contrast, no significant increase in apoptosis was observed in the p53 null cell line at this early time point (Figure 6A). Furthermore, MTT assays demonstrated reduced HCT-116 cell viability following both *RPL27A* and *RPS14* depletion. In contrast, *RPL5* depletion did not affect cell viability in either cell line (Figure 6B). Polysome profiling was performed on HCT-116 cells following *RPL27A*-sh2 infection (Figure 6C). Although *RPL27A* depletion reduced the 60S subunit, this was less marked than the later reduction observed on Day 6, suggesting that attenuation of the 60s subunit increases with time.

### *RPL27A* knockdown in normal CD34+ cells reduces cell proliferation and induces p53

**Figure 7:**
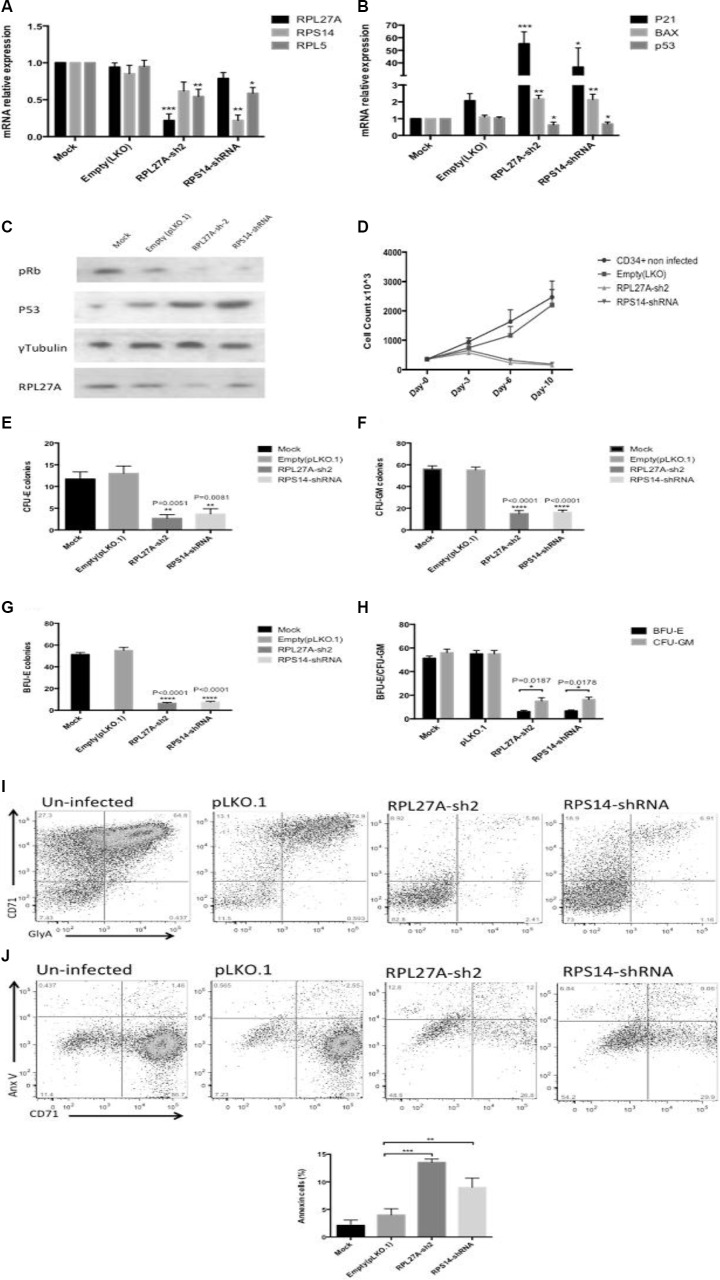
RPL27A knockdown in normal CD34+ cells blocks haematopoietic stem cell proliferation and differentiation Normal CD34+ cells underwent infection either with control, RPL27A-shRNA or RPS14-shRNA and samples collected for analysis on Day 6. A significant reduction in *RPL27A* and *RPL5 mRNA* expression was evident following *RPL27A*-shRNA infection. Reduced *RPS14* and *RPL5* expression followed *RPS14* shRNA infection (**A**). Upregulated expression of *p53 mRNA* and both *p21* and *Bax* was induced following either *RPL27A* or *RPS14 shRNA* infection (**B**). Depletion of *RPL27A* and *RPS14* also resulted in increased p53 protein expression and hypophosphorylation of phospho-Retinoblastoma (pRB) protein (**C**). Enumeration of cells at 48-hour intervals post infection demonstrated reductions in cell numbers following infection with either *RPL27A*-sh2 or *RPS14*-shRNA compared to controls (**D**). Methylcellulose colony formation assays were used to assess frequencies of progenitor cells following infection with either control, *RPL27A*-shRNA or *RPS14* shRNA. In both *RPL27A* and *RPS14* deficient cells, the most prominent reduction was in the erythroid lineage. Data is representative of 4 independent experiments, bars represent the mean ± SEM. CFU: colony forming unit including all colony types except erythroid; CFU-E: colony forming unit erythroid only (**E**–**H**). Flow cytometry was performed on cells harvested on Day 10/11 post infection. Cells were labelled with a combination of conjugated fluorometric antibodies including live/dead staining (780), an immature erythroid marker (CD71) and mature erythroid marker (Glycophorin A (GlyA)). Following either *RPL27A-shRNA* or *RPS14* shRNA infection, a significant reduction in erythroid cells was evident compared with controls (**I** + **J**).

For Peripheral Blood Mononuclear Cell (PBMC) derived CD34+ cells, both *RPL27A* and *RPS14* shRNA reduced the expression of *RPL27A* and *RPS14* mRNA respectively by ≈70% compared to control (Figure 7A) and RPL27A knockdown was confirmed by western blot analysis (Figure 7C). Despite an apparent decrease in *p53* mRNA expression in both *RPL27A* and *RPS14* depleted cells, there was an increase in both p53 protein expression and *p21* and *Bax* transcripts (Figure 7B and 7C). In addition, both *RPL27A* and *RPS14* shRNA attenuated cellular proliferation as demonstrated by hypophosphorylation of Retinoblastoma protein (Rb) and a reduction in cell enumeration (Figure 7C, 7D).

Next, we established the effect of *RPL27A* knockdown on erythroid cells. Erythroid differentiation was assessed on day 10 by flow cytometric expression of the erythroid- specific CD71 and Glycophorin A (GlyA) positive cells within the gated live cell population. Marked reductions in both immature and mature erythroid cells were evident in *RPL27A* and *RPS14* deficient cells respectively (Figure 7E). Apoptosis was assessed by Annexin V staining and confirmed increased apoptosis in erythroid cells (Figure 7F). Lastly, RPL27A deficiency significantly blocked erythroid and granulocyte/ macrophages (GM) colony formation as assessed by clonogenic assays. The reduction in erythroid forming colonies was the most prominent finding (Figure 7G–7J).

### *miR-595*, *RPL27A* and *RPS14* expression in MDS patients

**Figure 8:**
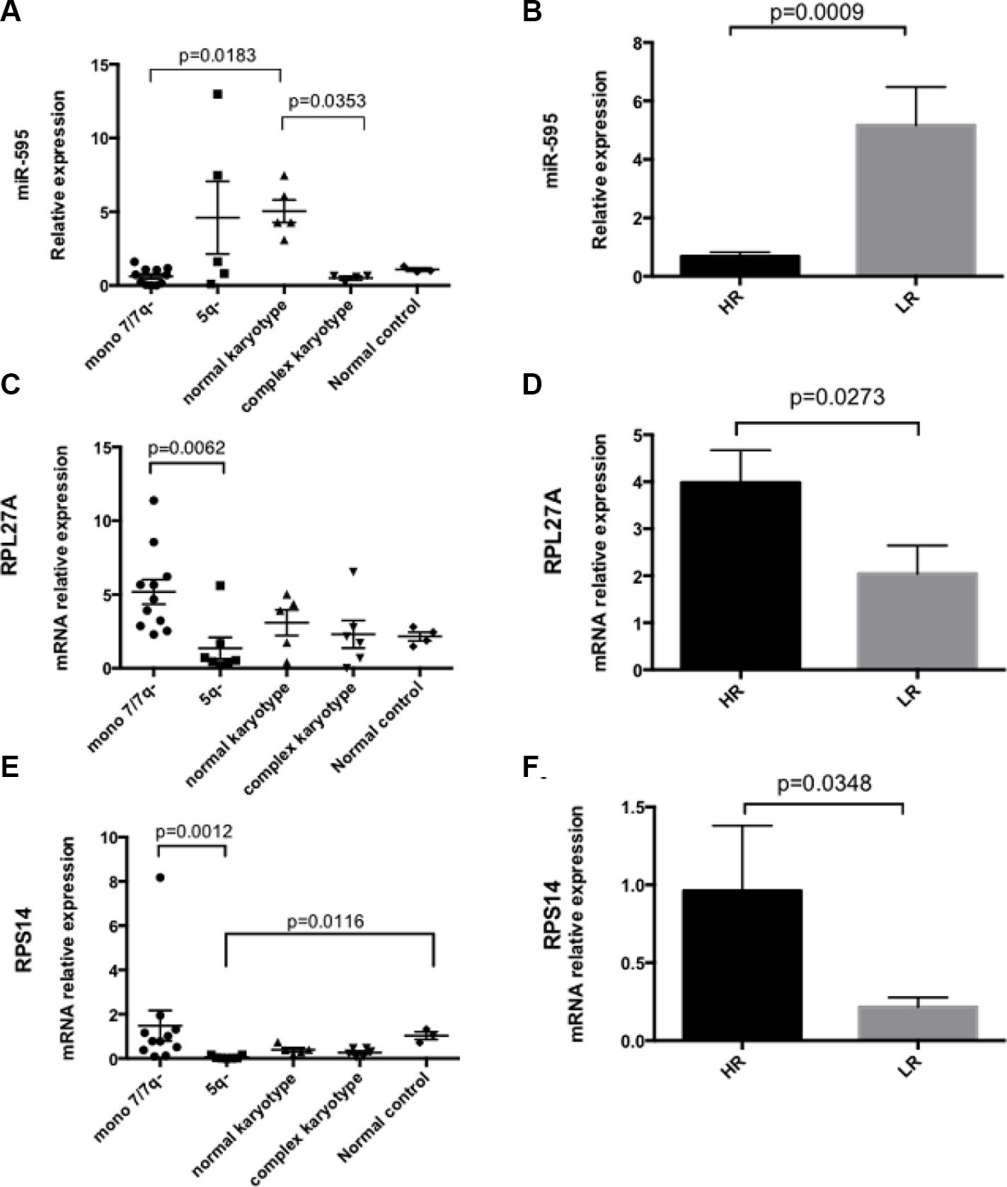
*miR-595*, *RPL27A* and *RPS14* expression in MDS bone marrow CD34+ samples First, *miR-595* expression was analysed in bone marrow CD34+ samples from 29 MDS patients (mir-595 was not detected in 2 patients). *miR-595* expression was significantly downregulated in MDS with −7/7q- MDS (*n* = 11) compared with normal karyotype MDS (*n* = 5). Significant differential expression of *miR-595* was also evident between CD34+ cells derived from MDS patients with complex karyotype containing chromosome 7 anomalies compared to those from MDS patients with a normal karyotype (**A**). *miR-595* was significantly upregulated in LR MDS compared with HR MDS (**B**). Relative expression was calculated using the ΔΔCT method and *RNU6B* was used to normalize all samples. Bars represent mean ± SEM. Next, *RPL27A*, *RPS14* and *RPL5* expression was determined in BM CD34+ samples from the same 29 patient cohort (**C**–**F**); Four control CD34+ samples were available. *RPL27A* was significantly upregulated in patients with −7/7q- compared with 5q- patients. Re-analysis according to IPSS score (HR = 17 and LR = 12) demonstrated that *RPL27A* and *RPS14* were significantly upregulated in HR MDS compared with LR MDS in this cohort. Bars represent mean ± SEM.

The relative expression of *miR-595*, *RPL27A* and *RPS14* in bone marrow CD34+ cells derived from 29 MDS patients was compared to 4 controls. Supplementary Table S4 displays patient clinical details. As regards cytogenetics, 11 patients had −7/7q-, 6 patients had a complex karyotype inclusive of chromosome 7 anomalies, 7 patients had del5q and 5 patients had a normal karyotype. *P53* mutational status was not available for all patients. The microRNA *miR-595* was detected in 27/29 patients and 3/4 controls. Of note, *miR-595* expression was significantly downregulated in MDS with −7/7q- MDS (*n* = 11) compared with normal karyotype MDS (*n* = 5). Furthermore, *miR-595* was significantly downregulated in MDS with complex karyotype inclusive of chromosome 7 anomalies (*n* = 6) compared with normal karyotype MDS (*n* = 5) (Figure 8A). Of note, in this cohort, *RPL27A* was significantly upregulated in patients with −7/7q- compared with 5q- patients. No significant expression changes were identified between the −7/7q- cohort and other cytogenetic groups. Lastly, we compared *miR-595* expression between IPPS High Risk (HR) and low risk (LR) groups. *miR-595* expression appeared lower in HR disease when compared to LR disease, accompanied by higher expression of both *RPL27A* and *RPS14* (Figure 8B–8F). These findings need confirmed in a larger cohort.

## DISCUSSION

Our novel assay identified several putative targets of *miR-595,* a microRNA residing on chromosome 7, including *RPL27A* and *HSPA14.* We hereby confirm, for the first time, that the ribosomal subunit *RPL27A* is a target for *miR-595*. Downregulation of *RPL27A* expression, to model haploinsufficiency, induced *p53-*mRNA and protein expression, accompanied by upregulation of *p21* and *Bax,* in the HCT-116 cell line and upregulation of *p21* and *Bax* in HEL cells. In contrast, in p53 null cells, expression levels of *p21* and *Bax* did not significantly change, implying that *RPL27A*, akin to other ribosomal proteins functions through p53-dependent mechanisms. This finding is consistent with a previous study investigating RPL27A in Sooty Foot Ataxia (SFA) mice [13–15, 17, 18, 25].

In our study, depletion of *RPL27A, RPS14* and *RPL5* reduced *MDM2* mRNA and protein expression in all cell lines except the K562 cell line. Moreover, we demonstrate that endogenous RPL27A interacted with both MDM2 and RPL5 in HCT-116 cells. This suggests that RPL27A may well, at least in part, be working through MDM2 to influence p53 expression. The BLAST alignment of RPL27A and RPL5 sequences resulted in two regional alignments with 85–89% identities, which supported the concept of possible binding between both moieties. Moreover, a computational based protein interaction application (BioGrid) predicted possible binding of RPL27A with both MDM2 and RPL5.

Sh-RNA mediated silencing of *RPL27A, RPS14* and *RPL5* reduced proliferation capacity in both HEL and K562 cells, suggesting p53 independent effects. Decreased proliferation was additionally confirmed in the U937 p53 null cell line. We also show that *RPL27A* depletion in both p53 functional and null cells is associated with increased apoptosis, suggesting both p53-dependent and independent effects.

It is well established that mutations or altered expression of ribosomal proteins may lead to dysregulated ribosome generation. We show that *RPL27A* knockdown via RPL27A-sh2 led to depletion of the 60s subunit accompanied by an increase in the 40s subunit. Our findings contrast previous work in SFA mice where no effect on ribosome biogenesis was observed [17]. However, less efficient knockdown of *RPL27A* in that experimental setting may well explain this differing result. In addition, we highlight, for the first time, the importance of *RPL27A* in maintenance of nucleolar integrity and role in ribosome synthesis/maturation. Previous groups have suggested no effect on nucleolar integrity following either *RPS14* or *RPS19* knockdown [26, 27].

Knockdown of *RPL27A* in normal CD34+ cells resulted in increased p53 protein expression but not p53 mRNA, akin to what was observed in previous studies investigating *RPS14* and *RPS19* knockdown [18]. One potential reason for this finding is a global inhibition of translation due to associated ribosome dysgenesis. Furthermore, reduction of *RPL27A* significantly attenuated cell proliferation and induced apoptosis. These observations were also seen with *RPS14* knockdown and were in agreement with other studies, linking ribosomal haploinsufficiency to p53 activation. However, our initial work, and that of others have demonstrated additional p53 independent effects of *RPL27A* knockdown [26–28]. Functionally, we show that *RPL27A* depletion impaired proliferation of CD34+ cells. Marked reductions in both immature and mature erythroid cells was evident in *RPL27A* deficient cells similar to the effect of RPL27 and RPS27 haploinsufficiency in DBA patients [29]. Of relevance, several other studies have described an upregulation of RPL27A during early erythroid development and hence suggest a pivotal role of RPL27A in erythropoiesis [30, 31]. For both −5q syndrome and DBA, mouse embryonic stem cell and zebra fish embryo models with depleted ribosomal proteins displayed anaemia regardless of p53 status, although we acknowledge the inherent limitations of these model systems [32–34].

Finally we demonstrated that *miR-595* was significantly downregulated in MDS patients with −7/7q- compared with patients with normal karyotype. Interestingly, *miR-595* was also significantly downregulated in MDS with complex karyotypes containing chromosome 7 anomalies and also when IPSS HR cases were compared to LR cases. Expression of *RPL27A* was higher in MDS with −7/7q- compared with 5q- MDS and also in HR-MDS compared with LR-MDS. These findings are in agreement with previous work correlating ribosomal protein overexpression with both disease aggressiveness and progression [35, 36]. The prognostic significance of *RPL27A* requires further validation in a larger patient cohort.

In summary we have described that miR-595, located on chromosome 7q36.3, is a pivotal miRNA regulator of RPL27A. *In vitro* we have demonstrated both p53-dependent and independent effects following *RPL27A* deficiency, including attenuated cellular proliferation, apoptosis and defective ribosomal biogenesis. It is evident that *RPL27A* depletion leads to a severe cellular insult, which appears to have a predilection for early erythroid cell development. We show that *miR-595* was significantly downregulated in MDS patients with −7/7q- compared with patients with normal karyotype. Given the chromosomal location of *miR-595,* at 7q36.3, the clinical relevance of these findings will require further exploration in the context of MDS patients who display chromosome 7 anomalies.

## MATERIALS AND METHODS

Methods not listed below are described in the online supplementary files

### Cell culture and transfection

Adherent cells (MCF7, HeLa, HepG2, HCT-116 and HCT-116 p53^−/−^) were cultured in Dulbecco modified essential medium (DMEM) media. Suspension cells (K562, KG-1, HEL and U937) were cultured in RPMI 1640 media. Both culture media were supplemented with 10% (v/v) Fetal Bovine Serum, 1% (v/v) L- Glutamine, penicillin-streptomycin, and 1% (v/v) sodium pyruvate. Cells were cultured under humidified condition at 37°C in a 5% CO incubator. The miRNA transfections and negative control pBabe transfections were performed using nucleofection technology according to the manufacturer's instructions (Lonza). The miRNA sequence is listed in Supplementary Table S1.

### Novel functional assay for miR-595 identification

The micro-RNA *miR-595* was cloned by PCR amplification of a ~1 Kb fragment obtained from human genomic DNA with primers incorporating the restriction sites *BamHI* and *EcoRI.* The resultant PCR fragment was cloned into pCR2.1-TOPO vector using pCRII-TOPO TA cloning kit (Invitrogen) and sequenced. Plasmid DNA was digested with *BamHI* and *EcoRI* and cloned into the retroviral vector, pBabepuro. The novel miRNA functional assay was performed as previously described (Gaken *et al.* 2012) The 3′untranslated region (3′UTR) cDNA library (MREH01, Sigma), derived from 10 different human tissues and 10 human cell lines and representing 16,923 unique genes, was cloned downstream of the *TKzeo* fusion gene into *Sfi1* sites in a p3′TKzeo vector. Isolated genomic DNA from the functional assay was amplified by PCR with p3′TKzeo specific primers flanking the cloning site (Forward GGGTCGACCTCGAATCCTTA and Reverse CGAGGCGGCCGACATGTTT). The PCR product was sequenced directly to identify targets for miR-595. Target identification and validation was applied as previously described (Gaken *et al.* 2012).

### Ribosome profiling

Cycloheximide was added to those cell lines under investigation to obtain a final concentration of 100 μg/mL followed by incubation in a tissue culture incubator for 15 minutes. Between 1–10 × 10 cells were subsequently harvested and washed with PBS containing 50 μg/mL Cycloheximide. Cell lysis, sucrose gradient centrifugation and polysome profiling were then performed as previously described (Fumagalli *et al*. 2009). Briefly, cells were lysed in polysome lysis buffer, incubated on ice for 15 minutes and centrifuged at 10,000 × g for 10 minutes at 4°C. Equal lysate, according to the Optical Density (OD), was layered on top of a 10–50% (w/v) sucrose gradient and centrifuged for 1 hour and 57 minutes at 40,000 × g at 4°C in a SW41Ti rotor (Beckman). Gradients were fractionated using the polysome fractionator and Polysome profiles signals were detected using a UV monitor at A_254._

### Patient samples

This cohort incorporated 29 bone marrow samples from patients with MDS, which underwent CD34+ selection. Normal CD34+ cell products were obtained from our Haemato-Oncology Tissue Bank (Human Tissue Authority; licence number 12223). Samples were used in accordance with ethics approval given by the UK National Research Ethics Service (NRES) (Approval Reference 08/H0906/94).

### Statistical analysis

Statistical analysis was performed using GraphPad Prism version 6 (La Jolla, CA). Data was compared using paired *t* tests. *P* values < 0.05 were considered statistically significant.

## SUPPLEMENTARY MATERIALS FIGURES AND TABLES


