# Supplementary material for: RPL27A is a target of miR-595 and may contribute to the myelodysplastic phenotype through ribosomal dysgenesis
Source: Oncotarget. 2016 Jun 25;7(30):47875–90. doi: 10.18632/oncotarget.10293 (PMC5216985; doi:10.18632/oncotarget.10293)
Supplement: Supplementary file 1 [file oncotarget-07-47875-s001.pdf]

RPL27A is a target of miR-595 and may contribute to the myelodysplastic phenotype through ribosomal dysgenesis

Supplementary Materials

ADDITIONAL METHODS

Lentiviral production and infection

Lentiviral shRNAs were incorporated in the pLKO.1 vector plasmid. *RPL27A, RPS14* and *RPL5* shRNA were obtained from Open Biosystem. Both the *RPS14* shRNA and 'pLKO.1 empty' vector were gifted by Dr. Jie Jiang, King's College, London. The corresponding shRNA sequences are listed in Supplementary Table S2. Adherent cells were seeded in 24-well plates at a density of 4–5 × 10^5 cells per well in 1mL of DMEM for 24 hours prior to transduction. Suspension cells were seeded at the same density in 1mL of RPMI 1640 and transduced on the same day. Viral particles (100 µL) were added to the cells followed by 8 µg/mL of polybrene. After 24 hours culture, the supporting medium was changed and infected cells was selected with Puromycin (2 µg /ml). Of note, the normal CD34+ cells were infected in a similar fashion but 10 µL of 100× concentrated lentivirus was used for infections.

Culture of primary cells and flow cytometry analysis

Peripheral Blood Mononuclear Cells (PBMCs) were isolated using Ficoll density-gradient centrifugation. CD34+ isolation was performed using a CD34+ MicroBead Kit following the manufacturers protocol (Milteny Biotech). The isolated CD34+ cells were cultured in two phases in liquid culture. For the first 6 days, cells were cultured in Serum-Free expansion medium (StemCell Technologies), supplemented with 100 U/mL penicillin/streptomycin, 2 mM glutamine, 100 ng/mL stem cell factor (SCF), 10 ng/mL interleukin-3 (IL-3), 10 ng/mL interleukin-6 (IL-6) and 0.5U/mL erythropoietin (EPO). On day 6, cells were collected for both RNA and protein extraction; the remaining cells were split for two different liquid culture differentiation systems. To induce erythroid and myeloid differentiation, 3 U/mL EPO, 15 ng/mL granulocyte colony-stimulating factor (G-CSF) and 40 ng/mL FLT3 was added. Cells were harvested on day 10 or 11 (day 4 of the differentiation cycle) and stained for subsequent flow cytometry analysis. For flow cytometry the cells were washed with Phosphate Buffered Saline (PBS). Cell pellets were resuspended in 1mL PBS and mixed with 1 µL of live/ dead stain (eFluor® 780) and incubated in the dark for 30 minutes. Next, cells were washed twice with FACS buffer and stained with the corresponding marker antibodies and incubated for 30 minutes in the dark followed by a further two washes. The resultant supernatant was removed and the cell pellet was resuspended in FACS buffer and analyzed by FACS scan.

Quantitative RT-PCR

RNA from cell lines was extracted using the standard TRIzol (Invitrogen) methodology. The RNA from CD34+ cells was purified using a RNeasy Micro kit (Qiagen) according to protocol. The first strand cDNA was generated from 500 ng of total RNA using SuperScript® VILO™ cDNA Synthesis Kit (Invitrogen) following the manufacturer's protocol. Quantitative RT-PCR was performed using a Universal Probe Library (UPL) from Roche. The PCR primers and compatible probes were designed using the LightCycler® Probe Design Software 2.0, available on the Roche website. Relative mRNA expression was determined using the ΔΔCT method. A list of primers and probes utilised is provided in Supplementary Table S3.

The miRNA reverse transcription and quantitative PCR was performed as previously described [2]. miR-595 and RNUB6 (endogenous control) primers were obtained from Applied Biosystems. cDNA was generated from 5ng of RNA using TaqMan miRNA Reverse Transcription Kit (Applied Biosystems). The qPCR was performed using Taqman Universal Mastermix (Applied Biosystem).

Western blot analysis and co-immunoprecipitation (Co-IP)

Western blot was performed as previously described (2). Immunoblotting was performed with the following primary antibodies; Rabbit anti-human RPL27A (C-term, Abgent) at 1:500 dilution, Mouse anti-human p53 (DO1, SantaCruz Biotechnology) at 1:500 dilution, Mouse anti-human MDM2 (2A10, Abcam) at 1:500 dilution and goat anti-human tubulin (C-20, Santa Cruz Biotechnology) at 1:1000 dilution, Phosph-Rb (Ser807/811, Cell signalling). Whole cell lysate from 6 × 10^6 cells were harvested and

co-immunoprecipitation was performed as described [26, 27]. IP assay was conducted using anti-human RPL27A (C-term, Abgent) and anti-MDM2 (2A10, Abcam).

Flow cytometry for apoptosis and cell cycle analysis

Apoptosis was assessed by staining with Annexin V and 7-anti actinomycin D (7-AAD) (Biolegend) according to the manufacturer's instructions. For cell cycle distribution analysis, cells were stained with propidium iodide/RNAse staining solution. Flow cytometric analysis was performed on a FACS Canto2™ platform and data was analysed using FlowJo™ software.

Immunoflorescence analysis of nucleoli structure

A total of 5×10^4 of the cells under study were re-suspended in 200 μL PBS and subsequently spun onto slides using a Cytospin. Following an Abcam immunofluorescence protocol, Anti-Fibrillarin antibody (Abcam, ab5821) was used at 1:200 dilutions in PBS supplemented with 1% Tween-20 (TPBS). Slides were then analysed via confocal microscopy to visualize nucleoli signals.

Methylcellulose colony assays

At day 6 post infection, cells were plated in methylcellulose media containing SCF, IL-3, IL-6 and EPO (MethoCult GF M3434; StemCell Technologies). Medium was layered on a 6-well tissue culture plate. The plate was incubated for 14-16 days in a humidified incubator with 5% CO_2 at 37°C to allow colony formation and subsequent assessment.

Cell viability and proliferation assays

Cell viability was performed using standard MTT assay techniques.

Cellular proliferation was determined at 48-hour intervals. Cells were mixed with Trypan Blue dye and loaded into a Haemocytometer (Neubauer cell counting chamber, depth 0.1 μl) to facilitate cell counting.

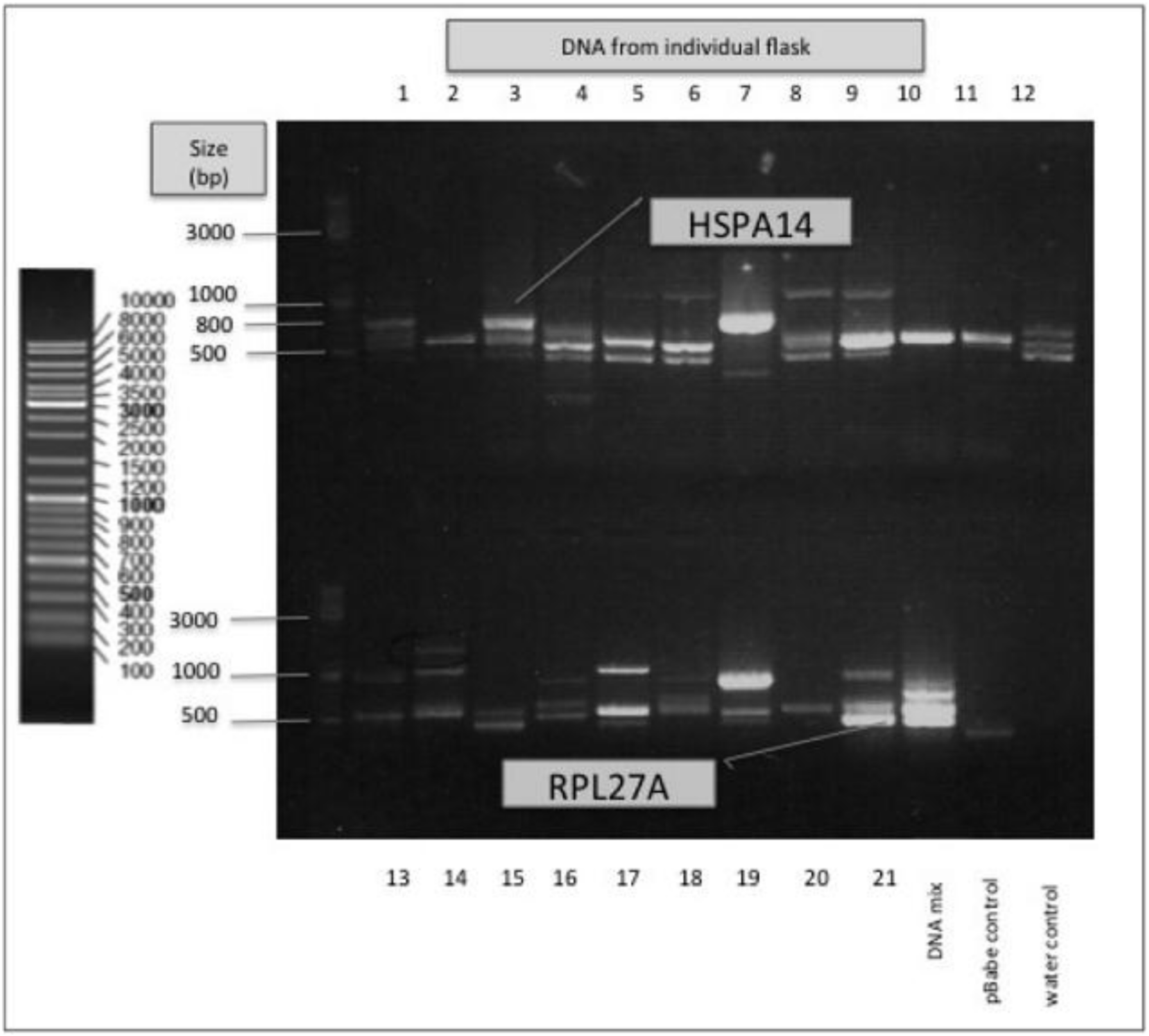

Supplementary Figure S1: Representative PCR amplification of extracted genomic DNA. The breast carcinoma MCF7 cell line, lacking miR-595, was transfected with cDNA library (Target ID Library, Sigma) and underwent zeocin selection. Expanded zeocin resistant cells underwent infection with either a pBabePuro vector expressing miR-595 or empty vector. After 48 hours, cells underwent puromycin selection and subsequent ganciclovir (GCV) counter-selection. Genomic DNAs were isolated from GCV resistant cells from 21 flasks and PCR amplified using vector specific primers. PCR products were visualized on a 2% (w/v) agarose gel and the gel image is representative of PCR products from individual flasks. Each lane represents DNA from one flask. Some of the bands are present in multiple samples and potentially represent identical targets in differing transfections. Bands of different sizes were hence purified and sequenced. A BLAST search of the sequences obtained identified two transcripts; RPL27a (≈ 500 bp) and HSPA14 (≈ 800 bp) as indicated.

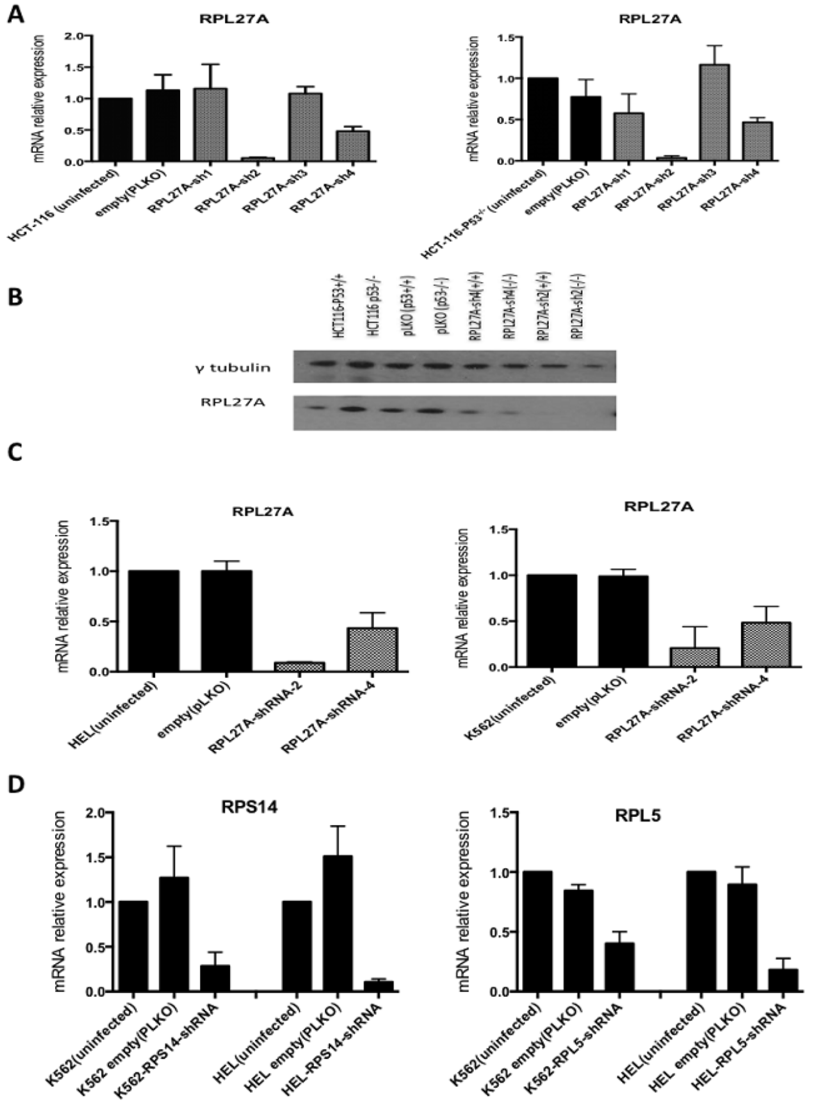

Supplementary Figure S2: RPL27A depletion using lentivirus short-hairpin RNA. RPL27A mRNA and protein expression was examined in HCT-116 and HCT-116 p53$^{-/-}$ cells infected with four different shRNAs to determine efficiency (**A** + **B**). Attenuated RPL27A mRNA and protein expression (using tubulin as a control) followed infection with *RPL27A*-sh2 and *RPL27A*-sh4 compared to controls. (**C**) RPL27A mRNA expression in HEL and K562 cell lines following infection with either *RPL27A*-sh2 and *RPL27A*-sh4 also displayed differential reductions in expression. (**D**) RPSA14 and RPL5 mRNA expression in HEL and K562 cell lines following infection with either *RPS14 shRNA* and *RPL5 shRNA* also displayed differential reductions in expression mRNA expression was normalized to GAPDH mRNA, calibrated to the non infected cells and compared with empty vector. Bars represent the mean ± SEM from three independent experiments.

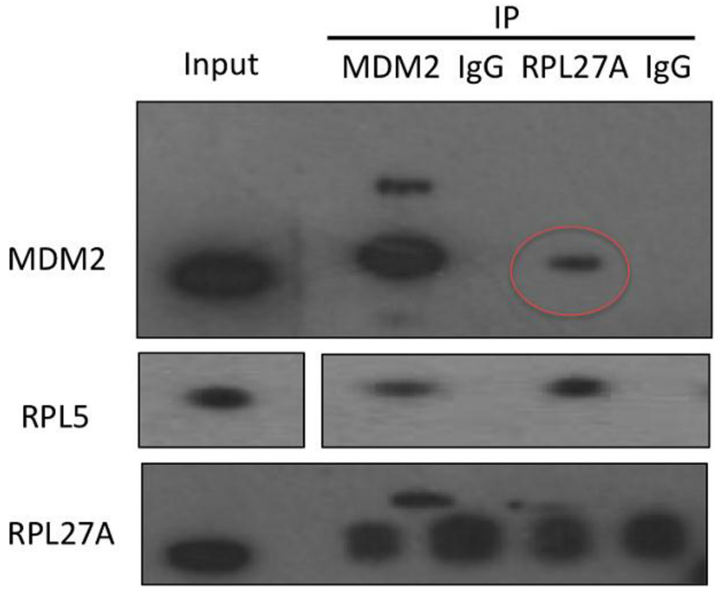

Supplementary Figure S3: RPL27A interacts with MDM2 and RPL5. Endogenous RPL27A interacts with endogenous MDM2 and RPL5 in HCT-116 cells as demonstrated by co-immunoprecipitation experiments. Cell lysates (5×10^6 cells) were prepared and immunoprecipitation was performed with anti-RPL27A or rabbit IgG and anti-MDM2 or mouse IgG followed by immunoblotting with anti-MDM2, anti-RPl27A and anti-RPL5. Experiments were performed at least in duplicate. This result demonstrates that MDM2 and RPL5 proteins are co-precipitated with RPL27A pull down.

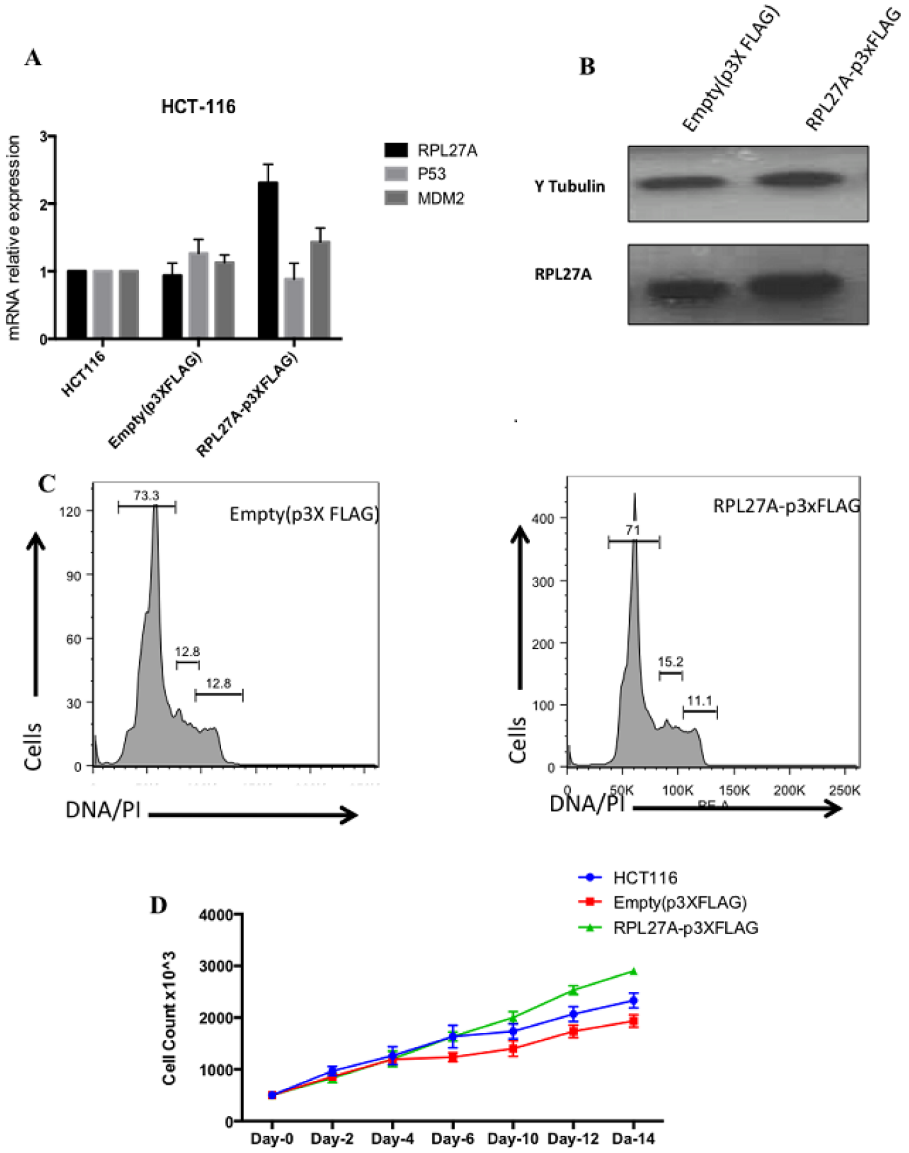

Supplementary Figure S4: Effects of RPL27A overexpression in HCT-116 cells. HCT-116 cells underwent mock transfection or transfection with empty vector p3X FLAG and p3XFLAG RPL27A constructs. Cells were selected with G418 for 14 days then collected to analyse RPL27A overexpression levels and effects on p53 expression and the cell cycle profile. The mRNA relative expression of *RPL27A*, *p53* and *MDM2* normalised to GAPDH in cells transfected with RPL27A compared with cells transfected with empty vector. Bars represent three independent experiments mean ± SEM (**A**). Western blot analyses of protein samples collected from cells transduced with RPL27A and empty vector. Samples were probed with RPL27A antibody and tubulin used as loading control (**B**). Cell cycle analysis of cells transfected with empty (p3XFLAG) and FLAG-RPL27A. Cells were fixed in ethanol and DNA content was stained with propidium iodidie and analysed by flow cytometry. No significant changes within cell cycle phases were identified from three independent experiments (**C**) Enumeration of cells at 48-hour intervals demonstrates increased cell numbers following infection with RPL27A-p3XFLAG (n = 3, mean ± SEM) relative to controls (**D**).

Supplementary Table S1: miR-595 and RPL27A oligomers

Oligo’s name	Sequence	Restriction enzyme
miR-595F	AAGGATCCAGGGTGCACTGAGGAAGATG	EcoR1
miR-595R	AAGAATTCGCCTCTGTCCATTCTCAAGC	BamH1
miR-595F2	AGCCATCAAGATGGTCTGC	EcoR1
miR-595R2	CCGGGACATGATGTGATTAG	BamH1
RPL27A-F	AGCTAGCAGGCCTTCCTTTTTCGTCTG	NheI
RPL27A-R	AGAATTCAATGAAACTCCCTCCATGTG	EcoR1

Supplementary Table S2: shRNAs oligomer sequences

shRNA Target	Sequence
Mature hsa-miR-595 Inhibitor	GAAGUGUGCCGUGGUGUGUCU
RPL27 lenti shRNA **(sh1)** TRCN0000117396	ATCGCACCACATCAATGATGG
RPL27 lenti shRNA **(sh2)** TRCN0000117392	TATTTGTCGAAGTTGATCCGG
RPL27 lenti shRNA **(sh3)** TRCN0000117395	TTCCCAGAACTTTGTAGTAGC
RPL27 lenti shRNA **(sh4)** TRCN0000117393	AACTTTCCCAAAGTAGCCTGG
RPL5 lenti shRNA **(sh1)** TRCN0000074993	TTTGTCACACGAACTATCATC

Supplementary Table S3: qRT-PCR oligomer sequences

Description	Sequence	Probe
RPL27A-F	5′-ACGGGTGAATGCTGCTAAAA-3′	32
RPL27A-R	5′-GAAGAATTTGGCCTTCACGA-3′	
RPS14-F	5′-GGTCCAGGGGTCTTGGTC-3′	17
RPS14-R	5′-GGTCCAGGGGTCTTGGTC-3′	
RPL5-F	5′-CACTGGCAATAAAGTTTTTTGGTG-3′	24
RPL5-R	5′-AACCAGGGAATCGTTTG-3′	
P53-F	5′-AGGCCTTGGAACTCAAGGAT-3′	12
P53-R	5′-CCCTTTTTGGACTTCAGGTG-3′	
CDKN1A (p21)-F	5′-TCACTGTCTTGTACCCTTGTGC-3′	32
CDKN1A(p21)-R	5′-GGCGTTTGGAGTGGTAGAAA-3′	
Bax-F	5′-AGCAAACTGGTGCTCAAGG-3′	69
Bax-R	5′-TCTTGGATCCAGCCCAAC-3′	
GAPDH-F	5′-AGCCACATCGCTCAGACAC-3′	60
GAPDH-R	5′-GCCCAATACGACCAAATCC-3′	
RNU6B	CGCAAGGATGACACGCAAATTCGTGAAGCGTTCCATATTTTT	NA

Supplementary Table S4: Patient characteristics

UPN	Karyotype	FAB	WHO	IPSS
1	45, XX,−7 [30]	RAEBt	AML	HR
2	45, XY,−7 [3] 46,XY [5]	RAEB	RAEB-2	HR
3	45, XX,−7 [5], 46 XX [15]	RAEB	RAEB-2	HR
4	45, XY,−7 [9]/ 46,XY [1]	RA	RCMD	VH
5	45, XY, inv (3) (q21q26), −7 [9]/46, XY [1]	RAEB	RAEB-2	HR
6	46, XY, −7, + mar [4]/45, XY −7 [3]/46,XY [8]	RAEB	RAEB-1	HR
7	45, XY, −7 [17]/46, XY [3]	RA	RCMD	HR
8	46, XY, +Y, −7 [6]/46. XY [3]	RAEBt	AML	HR
9	45, XY, −7 [3]/46, XY [7]	RAEB	RAEB-2	VH
10	45, XY, −7 [7]/46, XY [3]	sAML	sAML	H
11	LOH 7Q34–Q36.3	RAEB	RAEB-1	VH
12	COMPLEX	CMML	CMML	H
13	COMPLEX	RAEB	RAEB	H
14	COMPLEX	RA	RA	L
15	COMPLEX	AML	tAML	VH
16	COMPLEX	RAEB	RAEB-1	VH
17	COMPLEX	RAEBt	AML	VH
18	46, XX, del (5) (q13q31) [9]/46, XX [11]	RA	5q	L
19	46, XX, del (5) (q22q35) [13]/46, XX [2]	RA	5q	L
20	46, XX, del (5) (q31q31) [15]	RA	5q	L
21	46, XX, del (5) (q11q31) [9]	RA	5q	VL
22	46, XY, del (5) (q13q33) [11]/46,XY [19]	RA	5q	L
23	46, XY, del (5) (q13q31) [30]	RAEB	RAEB-2	VH
24	46, XX, del (5) (q13q31) [20]	RA	5q	L
25	Nk	RAEB	RAEB-1	L
26	Nk	RA	RA	L
27	Nk	RAEB	RAEB-1	I
28	Nk	RARS	RCMD	L
29	Nk	RA	5q	L
